# Benefits of a European Project on Diagnostics of Highly Pathogenic Agents and Assessment of Potential “Dual Use” Issues

**DOI:** 10.3389/fpubh.2014.00199

**Published:** 2014-11-11

**Authors:** Roland Grunow, G. Ippolito, D. Jacob, U. Sauer, A. Rohleder, A. Di Caro, R. Iacovino

**Affiliations:** ^1^Robert Koch Institute, Berlin, Germany; ^2^Spallanzani National Institute for Infectious Diseases, Rome, Italy

**Keywords:** EQAE in diagnostic, anthrax, tularemia, plague, melioidosis, glanders, brucellosis, dual use research of concern

## Abstract

Quality assurance exercises and networking on the detection of highly infectious pathogens (QUANDHIP) is a joint action initiative set up in 2011 that has successfully unified the primary objectives of the European Network on Highly Pathogenic Bacteria (ENHPB) and of P4-laboratories (ENP4-Lab) both of which aimed to improve the efficiency, effectiveness, and response capabilities of laboratories directed at protecting the health of European citizens against high consequence bacteria and viruses of significant public health concern. Both networks have established a common collaborative consortium of 37 nationally and internationally recognized institutions with laboratory facilities from 22 European countries. The specific objectives and achievements include the initiation and establishment of a recognized and acceptable quality assurance scheme, including practical external quality assurance exercises, comprising living agents, that aims to improve laboratory performance, accuracy, and detection capabilities in support of patient management and public health responses; recognized training schemes for diagnostics and handling of highly pathogenic agents; international repositories comprising highly pathogenic bacteria and viruses for the development of standardized reference material; a standardized and transparent Biosafety and Biosecurity strategy protecting healthcare personnel and the community in dealing with high consequence pathogens; the design and organization of response capabilities dealing with cross-border events with highly infectious pathogens including the consideration of diagnostic capabilities of individual European laboratories. The project tackled several sensitive issues regarding Biosafety, Biosecurity and “dual use” concerns. The article will give an overview of the project outcomes and discuss the assessment of potential “dual use” issues.

## Introduction

Internationally accepted biological infectious agents are divided into four risk groups based on their virulence, potential of public health threat, and availability of adequate treatment. Risk group 1 poses the lowest and risk group 4 the highest level of threat. A complex risk assessment for handling these pathogens leads to the definition of corresponding biosafety levels 1–4 (BSL1–4 or P 1–4) including technical, organizational, and personal protective measures. Highly pathogenic bacteria of risk group 3, e.g. *Bacillus anthracis, Yersinia pestis*, or *Francisella tularensis*, and risk group 4 viruses, e.g. haemorrhagic fever viruses, could cause severe diseases in humans and animals and are suspected to be used in bioterrorism attacks (1–7). Although there are various endemic areas in Europe for some of these zoonotic agents causing outbreaks, many questions about the epidemiology and ecology of these bacteria still remain open. In the context with other highly frequent diseases, the impact of infections caused by these bacteria and viruses on public health in Europe was so far rather limited. This also seems to be one of the reasons why the commercialization of diagnostic tests for these agents has not raised large interest and the microbiological laboratories are mostly forced to rely on their in-house assays. However, reliable diagnostics should be at hand for eventual natural outbreaks, for unpredictable imported cases and for the deliberate release of these agents, which poses an ongoing threat to the human population. Because of the different impacts and unpredictabilities of these agents to human health in different countries, networking of interested and/or appointed laboratories providing diagnostics in this field should be a logical consequence to exchange experiences, knowledge, and material supporting the laboratory response to outbreaks of these agents in single countries or cross-border events.

**Figure 1:**
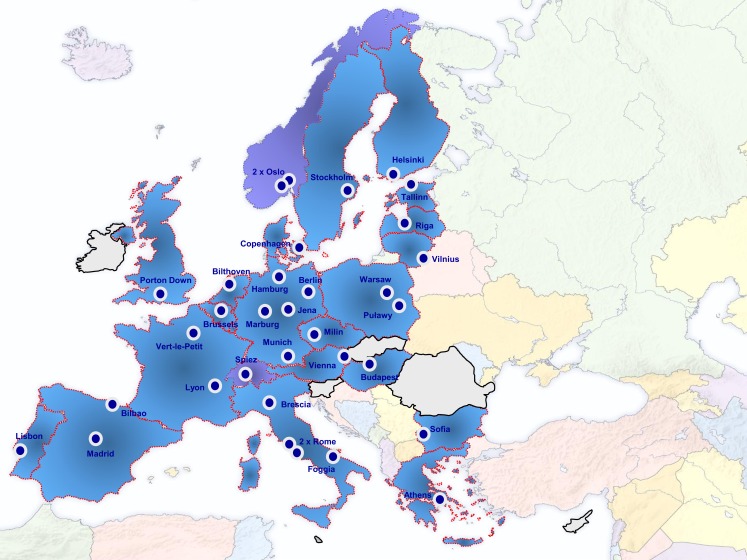
**Participating laboratories in QUANDHIP**.

Diagnostic laboratories need to participate in quality assurance exercises to assess their diagnostic approaches and to define measures for improvement and maintenance of their diagnostic capacities and capabilities. One of the reasons for the establishment of the EU Joint Action (JA) Quality Assurance Exercises and Networking on the Detection of Highly Infectious Pathogens (QUANDHIP) was the fact that capacities and possibilities to conduct proficiency tests in this field are very limited in many European countries (8). The JA is running from August 2011 to July 2014 and aims to link and consolidate the objectives of two existing networks dealing with highly infectious bacteria and viruses: The bacterial network emerged from the EU funded project EQADeBa (EAHC n°2007 204), coordinated by the Robert Koch-Institut (RKI), Germany, and served as a basis for the European Network on Highly Pathogenic Bacteria (ENHPB). The other one is the European Network of P4-laboratories (ENP4-Lab) project (EAHC n°2006 208), coordinated by L. Spallanzani National Institute for Infectious Diseases (INMI), Italy. The primary objective of the JA is to stabilize both network activities, which link 37 highly specialized and advanced partner laboratories from 22 European countries (Figure 1).

The overall goal of the project was the improvement of the detection and diagnosis of highly pathogenic bacterial and viral agents as well as to provide and further develop the laboratory support to the EU in the management of biological cross-border events. The range of target agents to be diagnosed is given in Table 1.

**Table 1:** **Diagnostic target agents in QUANDHIP**.

Bacteria	Viruses
*Bacillus anthracis*	Filoviruses (Ebola hemorrhagic fever)
*Francisella tularensis* ssp. the subspecies level	Arenaviruses (Lassa hemorrhagic fever)
*Yersinia pestis*	Bunyaviruses (Crim Congo hemorrhagic fever)
*Burkholderia mallei*	Orthopoxviruses
*Burkholderia pseudomallei*	Paramyxoviruses like Nipah and Hendra viruses
*Brucella* sp.	New viruses
*Coxiella burnetii*	

The JA developed a supportive European infrastructure and strategy for external quality assurance exercises (EQAEs) in order to establish a universal exchange of best diagnostic strategies. The EQAEs included shipment of infectious reference material, bacterial antibiotic susceptibility testing, the development of international repositories of reference material, shipment of living bacterial cultures, training, and Biosafety and Biosecurity reviewing of current practices. A special work package (WP) was directed to describe the capacities and capabilities of European laboratories, which are responsible for the analysis of highly pathogenic infectious agents and to provide recommendations on the activation mechanisms and the support offered by the QUANDHIP partners and the network in case of biological cross-border events. Most of these activities and the related data required an assessment of bio-risks in terms of Biosafety and Biosecurity including dual-use research of concern (DURC), which will be discussed in this article together with the most important outcomes of the project.

The general discussion on DURC was renewed when two studies on transmissibility of the avian influenza virus H5N1 got published (9). The dual-use problem concerning research is described on the coordinator’s (RKI) website as follows: “Research and development in the life sciences have crucially contributed to today’s progress and improvement of living conditions. At the same time, findings in the life sciences often run the risk of being misused to the detriment of society and environment. This “double applicability” of scientific findings is described as the “dual use dilemma.” The potential for misuse of scientific findings is especially obvious for research on pathogenic microorganisms and toxins: on the one hand, research results regarding transmissibility, pathogenesis, and genomics of pathogenic biological agents are indispensable to prevent the agents’ spread and proliferation and to enable or improve the treatment of infection and exposure to toxins. On the other hand, these results can also potentially be misused to cause harm to humans, animals, or plants (9, 10). It should be considered that not only biological agents as tools of research but also information on the outcomes of research activities could be categorized as dual-use dilemma. Appropriate Biosafety and Biosecurity measures and conventions can contribute to prevent the harmful side of biological research. Our project contains three horizontal (coordination, dissemination, evaluation) and five core (EQAE, repository, training, Biosafety and Biosecurity, support in cross-border biological events) WPs, which will be illustrated and discussed in terms of Biosafety/Biosecurity and DURC issues.

## Material and Methods

### WP 1–3 project coordination, evaluation, and dissemination

Before submitting the application for the JA, all potential partners were selected by an official “Letter of Intent” stating and confirming that these public or governmental institutes have been assigned the task to perform diagnostics on highly pathogenic agents under appropriate Biosafety and Biosecurity conditions. This was approved by the project controlling “Consumers, Health and Food Executive Agency” (CHAFEA). The JA was managed by the coordinator RKI and the co-coordinator INMI. A Steering Committee (SC), consisting of selected partners, had the function, in addition to the coordinators, to check the correct and timely implementation of the work program. An external Scientific Advisory Board (SAB), comprising representatives of the European Centre for Disease Prevention and Control (ECDC), the World Health Organization (WHO), and the European Commission, was set up for the evaluation of the project activities and gave advice for the optimization of contents and the course of the project in general. Both coordinators kept close contact so that all decisions were agreed between the coordinators beforehand, and, where necessary, including the SC, SAB, and individual partners. The JA comprised common and separate actions for the bacterial and viral network, the Network on Highly Infectious Bacteria (abbreviated here as NIB), coordinated by the RKI, and the Network on Highly Infectious Viruses/P4-Laboratories (abbreviated here as NIV), coordinated by INMI. Altogether, three joint meetings of both bacterial and viral networks, combined with an SAB meeting, and three separate meetings of each of the networks were organized. The meetings were used to share scientific and administrative information. The coordinators are running a public website and an internal workspace on a secure official server to share sensitive information. The project was presented on several scientific conferences/meetings, and a number of publications were developed. Besides the continuous internal controlling, the external evaluation of the project was performed by the CHAFEA, also including an external review of the interim report, and by the SAB.

### WP 4 external quality assurance exercises

#### Administrative preparation

The providers of EQAEs, RKI and PUM, prepared the samples and took care of quality assurance and shipment. The partners carried out the analysis of the samples due to the given parameters. The shipment of samples was realized by the selected shipping agency fulfilling all regulations for transportation of dangerous goods and national import/export regulations (10–16). The EQAEs were conducted separately for the highly pathogenic bacteria, including living and inactivated bacterial samples of risk group 3, and for the risk group 4 viruses, only including non-infectious nucleic acid from risk group 4 viruses so far. According to the Consortium Agreement a Material Transfer Agreement (MTA) was signed for each EQAE by provider and recipient.

Beforehand, all partners were asked to provide and confirm officially that they are entitled to handle risk group 3 and/or 4 agents, respectively, and carry out the work under appropriate Biosafety and Biosecurity conditions using a questionnaire developed in the framework of the previous projects EQADeBa and ENP4 and further optimized during this JA (WP 7). Partners who are not yet or currently not able to handle risk group 3 bacteria agreed to receive only sets of inactivated samples. The prepared living and inactivated samples were suitable for the application of different methods like molecular genetic methods, immunological methods, biochemical methods, or microbiological methods. In case of risk group 4 viruses, nucleic acid samples were only delivered to participants who practically have the possibility to further analyze positive samples under BSL4 conditions, having direct access to those laboratories, or having established collaborations and agreements with such laboratories. The sample design, preparation, and quality control of the EQAEs are described under the Supplementary material.

The data analysis was performed by the providers of the EQAEs and recommendations given for further improvement. QuoData was chosen as subcontractor and developed a new software for data entry and analyses, which has been used for the evaluation of the second and third NIB-EQAE (17).

For EQAEs on viruses, the procedure for the preparation of samples sent for these exercises was (1) amplification of the virus in cell culture; (2) inactivation by gamma irradiation (implying fragmentation of viral nucleic acid); (3) verification of inactivation procedure, no addition of PCR inhibitors; (4) testing of stability of the sample; (5) serial dilution and testing via (RT)-PCR and q(RT)-PCR. In some cases, human sera, spiked with the virus and inactivated as above, were used as testing material.

### WP 5 repository

To establish an international bacterial RG3 repository, a number of strains were provided by the participants to the RKI, who has been setting up and is keeping this repository. All strains were confirmed for their identity and phenotypic and molecular characteristics. DNA and inactivated bacteria were developed as reference material. According to the procedure for usage of the repository, which has been agreed by all participants beforehand, a limited set of material was delivered to partners on request. The repository was also used for the development of EQAE samples.

The BSL4-laboratories developed a list of key reference viral strains located at individual laboratories. The exchange of material between partners was agreed and regulated. For security and administrative reasons the exchange of “living” risk group 4 viruses was reduced to a minimum.

### WP 6 training

From the very first beginning of the project, several training programs, usually running for one week, were designed by the participating laboratories and listed and made accessible to all partners by the coordinator first by e-mail, later via the internal workspace. Partners could select and prioritize training programs of up to 10 days they considered most beneficial. For security and administrative reasons, usually only staff listed in the grant agreement was allowed to attend the training.

For risk group 4 viruses, rather theoretical courses were organized, covering various aspects of BSL4 work (basic knowledge, biosafety, management issues, competency, and scenario exercises).

### WP 7 biosafety, biosecurity

The infrastructure checklists for Biosafety and Biosecurity composed of the two existing networks (EQADeBa/ENHPB and ENP4) dealing with highly dangerous bacteria and viruses, respectively, have been compared, evaluated, reviewed, and exchanged.

In addition, the checklists and recommendations produced by other already completed European programs [e.g. Biosafety-Europe, European Training in Infectious Disease Emergencies – ETIDE (18), European Research Infrastructure on Highly Pathogenic Agents – ERINHA (19)] have been assimilated for review and impact on the outputs of this WP. This work has been supported by an external internationally recognized specialist for Biosafety and Biosecurity.

### WP 8 support to cross-border events

In order to comply with the objectives, the Project Coordinators created an expert working group, composed of both Coordinators, supported by external expert consultants and by staff from European Health Authorities involved in the SAB of the project. The aim was to develop an operational document containing recommendations on laboratory management of biological events and defining the role and activation procedures for the QUANDHIP network in case of international biological cross-border events. The tasks were agreed with the representatives of the EC and worked out with the support indicated above. Possibly occurring real events will be used for the evaluation of the developed document.

## Results under Special Consideration of Security Issues

### Managing issues related to the sharing of sensitive information (regarding WP 1-3)

The QUANDHIP JA activities require the sharing of sensitive information between the consortium partners. This is made possible by strong joint project coordination (RKI and INMI) and a culture of trust that has been built over many years through various European networking projects in the area of high containment laboratory work. In addition to a core coordination, the QUANDHIP partnership relies on the inputs from a SC, consisting of the main activity leaders and an external SAB. To manage the collaborations within the consortium, collaborating partners, the SC, and the SAB, a number of specific agreements have been developed and signed by all parties. These agreements were approved by the legal departments of the coordinator and all participants. The agreements, which were signed by all partners, contained beyond administrative and financial issues several regulations also preventing any misuse of material, data and information like

– responsibilities of partners,
– guidelines for a reference material repository of highly pathogenic bacteria,
– conditions for distribution of highly pathogenic viruses,
– training, including security instructions or security check of personnel if required,
– non-disclosure of information,
– handling of data, dissemination, intellectual properties,
– Material transfer agreement (MTA) concerning the extension of the ENHPB repository, concerning the distribution of material from the ENHPB repository,
– model MTA for distribution of highly pathogenic viruses.

The network websites and electronic mail transfer have ensured regular communication between participants (8). The internal website was provided by a secure German official provider.

For external communication beyond the consortium, it was important to establish a communication channel and coordination with other relevant European networks like the ECDC funded project European Network for Diagnostics of “Imported” Viral Diseases (ENIVD) (20) and the ERINHA to improve collaboration and exchange of relevant information and to avoid duplication in any international activities. To achieve this, bilateral meetings were arranged and letters of collaboration developed in order to clarify how information would be exchanged in a responsible manner. In addition, a dissemination plan was developed and all QUANDHIP JA deliverables were assessed in order to determine their confidentiality level. Except for the detailed interim and final reports, which were restricted only to relevant EU stakeholders (defined by CHAFEA), most of the deliverables were supposed to be made available to the scientific community. As a matter of course, the detailed results of the EQAEs provided by the individual partners were treated confidentially by the evaluators of the EQAEs and were published only in an anonymized form. The primary target groups to be considered in the dissemination strategy were laboratory workers of the associated and collaborating partners dealing with the diagnostics of high threat pathogens, biosafety experts, first responders, clinical staff, and security forces. In the framework of this project, various documents including recommendations for diagnostics, Biosafety and Biosecurity, management of biological events, and risk assessment from the laboratory perspective were or will be developed until the end of the project. So far, 40 scientific presentations and publications were used for dissemination of information on the QUANDHIP JA.

The evaluation of the project was done by CHAFEA and reliable external specialists working together in the SAB who approved by their signatures the confidentiality rules and procedures of the Commission. Altogether, the internal and external management of information sharing benefits from a trusted network of experts, collaborating external partners, and transparency in terms of the documentation of the “code of practice” when information is produced through the work of the QUANDHIP JA. Ultimately, the management of dual-use risks associated with information sharing on these high containment pathogens is ensured through a strong project coordination team and close collaboration with the funding authorities.

### Dual-use issues concerned with “external quality assurance exercises” (regarding WP 4)

External Quality Assurances are part of a methodology aimed at assessing the quality of laboratory diagnostics and strategy at the participating laboratories. EQAEs can be helpful to identify ‘best practices’ for certain diagnostic approaches which can be exchanged between participants for improvement of own procedures. It also can be a reflection of the overall quality management systems of the laboratory. Within the QUANDHIP JA framework, EQAE rounds involve the preparation and shipment of a panel of coded samples (living or inactivated) to participating laboratories. The laboratories test the samples and then return the results to the central coordination place of the EQAE round. The coordination processes the results and provides feedback to all participants so that they can only identify their own proficiency scores but also in a way that they can compare it with the performance of all other participants in an anonymized way. This information enables the laboratories, which did not perform so well or have certain deficiencies, to perform internal checks of their diagnostic assays and quality systems and make adaptations to improve their performance in the next testing round. Also there is the opportunity for exchange of information, protocols, and event training of the partners to improve their proficiency. As this exercise involves the shipment of materials with dual-use potential and the reporting of sensitive information that could expose vulnerabilities in the capability to detect high containment pathogens, the QUANDHIP JA has developed strategies to mitigate this risk.

The results and lessons the QUANDHIP coordination team has learned from the experience of performing six EQAs (both within the NIB and NIV sub-networks of the QUANDHIP JA) are summarized here.

The first mitigation of risk was to ensure that the partners involved in the performance of the EQAs had the bio-risk management elements in place to receive non-attenuated or live strains of the pathogens belonging to the testing panel.

The agreed EQA objective was therefore to identify progress and best practices in the performances of the participating laboratories as well as to identify gaps to be filled. This rationale was of clear public health benefit for preparedness and strengthening capacity of laboratory response and, therefore, these benefits outweighed the risks of not performing such proficiency testing activities.

During this evaluation we are analyzing the exercise elements

– Shipment
– Response time
– Correct qualitative and quantitative results.

The bacterial EQAEs were focused on *B. anthracis, Y. pestis, F. tularensis, Burkholderia pseudomallei, Burkholderia mallei, Brucella melitensis*-group, and only in Q-2 on *Coxiella burnetii*. From 7 to 15 samples, according to the exercise scenario, containing living bacteria (native samples), partially mixed with typical “contaminating” bacteria and inactivated bacteria in a variety of complex matrices and were provided by the RKI. Typical composition of EQAEs and summarized results are given in Tables S1–S8 in Supplementary Material.

Shipment was identified as a potentially very sensitive element in the EQAEs and was therefore prepared with a comprehensive effort involving the provider as sender, shipping agency, and the consignee considering the international regulations for air (IATA) and ground (ADR) transportation of dangerous goods of class 6.2.

According to the European Export Regulations of goods with dual-use potential (10), the provider has received a general export license for the set of defined biological agents relevant for this project from the German Federal Office of Economics and Export Control (BAFA), which has fully implemented the European Regulation article 9 (2) (10, 21). This license is required for EU exports to any non-EU Member States. Anyway each EQAE has been announced to the BAFA and any additional national regulations of the EU Member States have been followed by the partners receiving the material.

It was extremely important for Biosafety and Biosecurity reasons to select a reliable shipping agency. In this context, a market analysis was conducted and revealed only one appropriate provider, who could be identified as an appropriate shipper in terms of shipment quality and bio-risk management. At the beginning of the project, some partners intended to use alternative shipping approaches for ground transportation to reduce costs. However, it appeared that the cooperation with less experienced companies caused doubts concerning an appropriate risk management with all involved parties (sender, shipper, and consignee), and the reduced costs were compensated by an increased work load. Most problems appeared through inadequate knowledge of drivers and equipment of vehicles according to the ADR and through the deficient traceability of sent material. As a commonly agreed solution it was decided to use only certified and known shippers. This approach resulted in a high quality of shipment without any lack of traceability. Some rare delays at custom authorities were usually due to lack of provided information by the consignees and technical problems at the customs office and could be solved by the support of the shipper. During the project, all participants were learning from these administrative issues and cross-border problems did not really occur.

This was part of the reason why the deviation of time, required for the delivery of samples to the various participants, was significantly reduced. All in all, the transportation time was relatively short and border issues were an exception and could be solved without serious problems.

It becomes very obvious that the involved laboratories substantially improved their response time by about 70%. This important improvement is due to a better preparedness of the laboratories through training effects during the exercises and an improvement of diagnostic algorithms and methods. This practically relevant achievement is a very impressive outcome of the project and is rather discouraging for a third party who might have the intention to misuse a time delay in diagnostics of the agents. On the other hand, this information might be helpful for other laboratories to adapt their own procedures to this high level standard.

The submission of correct quantitative and qualitative results and the assessment of good performance of the laboratories are a major element of EQAEs. In addition, even more profound analyses have been performed to identify best practices for correct and to reveal reasons for incorrect results (data not shown), which were discussed with the participants. This included applied algorithms and methods. Important conclusions could be drawn and related recommendations for improvement could be given, where appropriate.

As a conclusion, PCR or immunological approaches were identified as best practices for sample analyses as a first step for preliminary identification of target bacteria, which should be followed by a confirmation by cultivation/isolation of bacteria and subsequent identification of growing germs by PCR or other applicable methods like MALDI-TOF (22).

From our study it can be concluded that the range of results on the quantitative reference samples is by far too broad.

All in all, it can be stated that the laboratories performed on a high level of diagnostic quality. However, if the sample composition varied and got more complex and challenging, the individual as well as the overall results fell off in quality. Together with the revealed problems that occurred in terms of correct identification of *Brucella* species and *F. tularensis* subspecies, the detection of “mixed” samples, the quantification of target bacteria, and the antimicrobial susceptibility testing (data not shown), quite sensitive information has been produced. As a conclusion of the results, topical working groups were set up in the framework of NIB profoundly tackling AST, MALDI-TOF, and the development of quantitative reference materials. As done here there is a need to provide the scientific and health community with technical data on these issues to generate scientific and administrative input and support. Regarding AST, the European Committee on Antimicrobial Susceptibility Testing (EUCAST) is showing interest in our activities and a close contact could be established with the aim to approve our results and take those into consideration for the development of appropriate European standards. It can be assumed that this high standard of diagnostics is rather hindering a third party to misuse this information. On the other hand, the gained experience can be very helpful for other laboratories to improve their diagnostic approaches. Thus, without disclosing individual laboratory performances it seems to be of benefit to publish these data for the scientific community and policy decision makers in this area.

### Development and sharing of a repository of high containment pathogens and materials derived from it (regarding WP 5)

To perform EQA exercises and to validate existing and new diagnostic methods the consortium needs access to appropriate biological materials (i.e., living and inactivated strains, antibodies, reagents, etc.). During the previous EU projects EQADeBa and ENP4-Lab repositories have been established which could be extended and characterized as part of the QUANDHIP JA activities. One key question related to dual-use issues was how the information of the repository contents would be communicated and how requests from the different consortium members, collaborating partners, and external experts are dealt with. Internal data bases are available only on the restricted QUANDHIP JA workspace providing all available characteristics of the samples. An appropriate “MTA” for guiding the sharing and the use of strains has been developed and implemented (an example is provided as supplementary material). As for the EQAEs this MTA has been agreed between all partners beforehand including their legal departments. The MTA is intended to be used also for a rapid exchange of material in outbreak situations and as well for managing any use of the materials for subsequent research activities. Another use of the MTA is to advise good practices and to record the sharing of the materials as part of the bio-risk management aspect. All partners have been asked to provide relevant and characterized bacterial isolates and, both clinical and environmental samples. A procedure and recommendations for the transport of infectious material were developed, including the strict advice for consideration of national and international regulations, and according to the dangerous goods regulations.

Today, the QUANDHIP JA NIB repository consists of 148 different strains. From most of the bacterial isolates reference material has been produced as genomic DNA, as heat inactivated cells for non-spore-forming bacteria, as PAA inactivated cells for spore-forming bacteria, and as viable cells. This approach will be completed for all relevant strains. Moreover, further methods for inactivation, like gamma irradiation (23), and for storage of the material, like lyophilization, are under development. In the QUANDHIP JA NIV, the BSL4 laboratory partners have their own repository of viral agents are collaborating with the DG RTD funded European Viral Archives (EVA) project to support the quality, management, and distribution of viral strains and reagents (24).

In order to facilitate the safe and secure circulation of biological reference materials and procedures within the network, the following activities are in planning:

(1) to further develop a list of key reference strains of all BSL4 viruses within the participating members’ laboratories which has been established in a previous project;
(2) to promote the exchange of all reference strains of all BSL4 viruses with accompanying memorandum of understanding on use and dissemination where appropriate. National and international regulations as well as appropriate agencies have to be identified and considered for transfer of material assuring security and traceability;
(3) to exchange SOPs and supporting cells/reagents to facilitate the growth of all reference strains in each member’s laboratory, and
(4) to exchange SOPs for the molecular detection and specific identification of all key reference strains in all members’ laboratories.

In addition, it was also planned to develop and verify quantitative nucleic acid standards for the comparison of different methods and instruments.

### Continued professional development toward a competent and responsible workforce (regarding WP 6 and 7)

Partners of the network have offered practical laboratory based training to other partners covering laboratory diagnostic response strategies in terms of preparation and analysis of samples within BSL3 and/or BSL4 facilities focused on best practices. Twelve training courses have been provided by selected partners at their institutions. All associated partners have agreed on the course contents and defined learning objectives and intended outcomes. The evaluation of the training courses, provided so far, has shown the high benefit for the participants, which has been implemented for the optimization of laboratory practices for both diagnostic and bio-risk management.

This exchange of experiences is one major aspect for the improvement of the performances shown during the EQAEs and for the setting up of new technical approaches. Another result of these courses is that partners may tackle questions, e.g., of Biosafety and Biosecurity, on more familiar grounds, and find help by personal contacts in cases of emergent biological situations. From the perspective of dual-use one could argue that trainees get deep insight in best practices, capabilities, and capacities as well as security approaches on the trainer’s side. However, all the staff were selected and responsible for laboratory bio-risk management in the partners’ states. Moreover, we could create a culture of mutual trust and responsibility.

The training course within the NIV was organized in five sessions, covering various theoretical aspects of BSL4 work (basic knowledge, biosafety, management issues, competency, and scenario exercises). In addition, practical training was offered on BSL4 working conditions, handling a BSL3 glove box, sample storage, differential diagnosis of hemorrhagic fever viruses, and decontamination.

### Toward harmonizing bio-risk management practices (regarding WP 7)

For the development of operational Biosafety and Biosecurity recommendations useful for self-evaluation and to be agreed among the project consortium, the work was designed to identify, agree, and disseminate key elements of structure and operation of primary and secondary containment, building design and infrastructure, integrated special equipment, disinfection strategies, biosecurity issues, etc. The previously developed check lists were compared with guidelines and recommendations derived from documents produced by EC, WHO, CDC, CEN workshops and national authorities. Based on the practical requirements of institutions running or developing high containment laboratories, BSL3 or BSL4 check lists will be further developed and validated considering external input. Currently, this is under further development.

Considerable collaboration and input has been provided by the European ENP4 laboratory and Biosafety (EBSA, ECDC) community to the development of the CWA 16393: 2012 (25) – Laboratory bio-risk management-Guidelines for the implementation of CWA 15793:2008 Laboratory Bio-risk Management Standard.

The check list is of almost general character and avoids any detailed description of the bio-risk management. This information belongs only to the partner of the project and can therefore not be misused by third parties if not disclosed.

### From theory to practice – implementing laboratory response support according to the QUANDHIP core competencies (regarding WP 8)

All efforts and outputs of the JA are used to develop proposals to support the coordination of response to cross-border events with highly infectious pathogens is addressing the following areas:

– providing laboratory support for risk assessment in case of cross-border events with highly infectious pathogens;
– transportation of samples;
– development of collaboration models between specialized laboratories as well as microbiological and routine labs in order to better coordinate the response and to overcome problems due to different levels of technical equipment and knowledge;
– promoting interactions within the bacterial and viral networks;
– supporting cooperation models with Emergency services, clinical settings and Public Health officials (including the development of SOPs for handling of samples from first responders to BSL3/4 labs);
– developing secure laboratory procedures in case of intentional release, bridging CBRN investigation and forensic laboratory operations.

A document, including a set of recommendations for all mentioned issues, has been drafted, disseminated to and discussed with all project participants. In addition, links with other European initiatives in the field of mobile lab, like DEVCO, have been considered. Moreover, the final version of the document will be disseminated through the project website and via scientific and project meetings (inviting, e.g., EpiSouth plus (26), ERINHA, ENIVD).

## Discussion

It is now widely accepted in the scientific community to perform an assessment of the “dual-use” character of scientific projects. QUANDHIP is a JA aimed to perform quality assessments and improvement of the diagnoses of highly pathogenic bacterial and viral agents. Consequently, this also includes all measures for an appropriate bio-risk management considering the potential dual-use character of certain elements of the project. These elements consist of sharing sensitive information on diagnostic approaches, performance of diagnostic laboratories dealing with highly pathogenic microbiological agents, repositories of these agents, laboratory bio-risk management and, in addition, exchange of highly pathogenic biological agents between participating laboratories was practically performed.

Several research institutions and authorities, like the Robert Koch Institute, have developed a policy and recommendations for this assessment (27). The RKI policy on DURC which is the basis for the assessment of our projects and which is in line with officially published recommendations includes several criteria for the evaluation of research and development:

- to achieve transmissibility of microorganisms or to enhance their infectiousness,
- to increase the virulence of microorganisms or toxins,
- to increase the tenacity of microorganisms or toxins,
- to facilitate the intake of toxins,
- to promote or induce the resistance of microorganisms toward therapeutic or prophylactic antimicrobial or antiviral substances,
- to enhance the capacity for spreading or for easy release or making them “weapons-grade”,
- to weaken the response of the immune system against microorganisms,
- to alter the host tropism of a microorganism or a toxin,
- to increase the susceptibility of host organisms,
- to generate entirely novel pathogens or to recreate pathogens that had previously disappeared or had been repressed (eradicated/eliminated/controlled/vanished naturally),
- to alter the absorptive characteristics of a biological agent or the toxicokinetics in a manner that enhances their effect,
- to reveal methods to lower the effectiveness of medical countermeasures (vaccinations, therapeutic and prophylactic means),
- to hinder or prevent diagnostic procedures

In addition, other policies, like those provided by the U.S. National Science Advisory Board for Biosecurity (NSABB) and EU European Export Regulations, include a principal list of agents and toxins which might be misused with high consequences for public health in addition to specific aims of research projects (10, 28, 29). The target pathogens focused in QUANDHIP are listed in these lists of agents to be controlled for export.

Using this set of criteria we assessed the JA QUANDHIP for elements of DURC and, when relevant, how it was prevented. The sensitive scientific procedures in general mentioned in both RKI and NSABB documents lead to the conclusion that no potential for DURC can be identified although, in contrast, the indicative list of agents published by the U.S. NSABB, the exchange of these materials between laboratories and the handling of sensitive information are raising the need to consider potentially DURC. However, the work of our project is not focused on research of these organisms but rather on best practices to detect and identify these microorganisms. Several international guidelines, regulations and recommendations as well as our own responsibility lead to the inevitable conclusion that this project is sensitive in terms of Biosafety and Biosecurity and measures had to be undertaken to prevent misuse of infectious agents and of information generated and used in the framework of this project. Under this premise we would like to illustrate some relevant issues in the project.

Most relevant in this context might be WPs 4 (EQAEs) and 5 (Repositories) where we exchanged highly pathogenic inactivated and living microorganisms between participating laboratories. Beforehand, we developed a strategy to minimize the risk of misuse or loss of these agents as already described in the previous sections. The strategy included

– appropriate selection of participating governmental laboratories and written confirmation by national governments and the European Commission
– official signature of a restrictive legally checked Consortium Agreement and MTA
– approved comprehensive check list for laboratory Biosafety and Biosecurity developed basically in the framework of a previous project (e.g., 180 check points for BSL3 laboratories)
– substitution of fully virulent pathogens by attenuated microorganisms or non-infectious material where possible
– selection of a reliable and certified shipment agency with an approved biosecurity policy and fulfilling the international regulation for transportation of dangerous goods
– consideration of national and international border regulations for import and export of infectious agents based on the Australia Group recommendations and Common Control Lists (30)
– and not to be underestimated: the emergence of trust and openness between participants by getting to know each other during regular meetings and training courses.

Taking this preparation into account three EQAEs including living bacteria for NIB and three EQAEs with inactivated material for NIV were carried out during the project. These exercises were technically most challenging for the participating laboratories. Together with a gap analysis of methods and procedures the EQAEs and training courses led to a substantial improvement of the performance of many laboratories. Collaboration between laboratories formed the basis for mutual support in emerging biological cross-border events but also for scientific collaboration. The provided material was also used as a means of assessment for further evaluation and improvement of laboratory techniques. Several participating laboratories were using the results of the EQAEs for accreditation purposes. In this respect, the QUANDHIP project had an exceptional international dimension because the organization of such EQAEs at national level is almost impossible and not cost-effective due to a too low number of participants. The check lists for Biosafety and Biosecurity were used by participants for self-evaluation but also in some cases for evaluation of newly constructed high containment laboratories.

The gained experiences regarding shipping issues will also serve sample sharing during an acute outbreak situation. All samples delivered in the framework of this project were shipped according to international regulations for the transportation of dangerous goods (12, 13). In addition, national regulations for import and export of such material were considered and a certified shipping agency contracted. Consequently, no serious problems occurred in terms of transportation of the samples, nor during border transfer due to a well prepared action.

Most EU Member States do probably not carry out proficiency tests for diagnostics of highly pathogenic infectious agents at national level although this would be strongly required to ensure the necessary quality of diagnostics (30–32). It should be considered that all activities described above are not only important from the perspective of intentional release of these highly pathogenic microorganisms as these bacteria also occur naturally in the environment and require appropriate diagnostic tools on a regular basis (33–38). The European scope of the project offers the appropriate framework to evaluate, improve and sustain these diagnostics to have a broader platform to develop and exchange knowledge, methods and reference material on these often “neglected” but potentially very dangerous diseases.

Not only agents but also information could be misused. On the one hand, this was mainly prevented within the consortium by signed agreements, on the other, a dissemination plan was developed and the exchange of sensitive information was realized using a specially secured German governmental internet provider. The internal workspace was continuously administered and moderated by the coordinator. The individual results of the EQAEs were strongly confidential and visible for the single participant only. Only a restricted number of persons from the coordinators´ part could see all results and draw anonymized overviews and trend analyses. There is no other way than by the laboratories themselves to make their individual results available to the public. Confidentiality is not only important because of the good laboratory practice but also because of security issues, i.e., to prevent information on gaps in the analytical capability of a certain national laboratory which could be intentionally misused by third parties. In contrast, overviews on best practices and possibilities of optimization of diagnostic approaches, published in scientific journals and reports, and presented on scientific meetings, could be useful for non-participating laboratories and stakeholders. The more detailed reports of the project were restricted to CHAFEA for defining the further usage.

Also the recommendations and description of the activation procedure of the QUANDHIP network in biological cross-border events, drawn in WP 8, could be regarded as sensitive information. This document also includes a list of national laboratories appointed to perform diagnostics on highly pathogenic infectious agents. This operational document should be used by EU and national decision makers and be distributed to other laboratories conducting “first line” diagnoses. The negative side of the “dual-use” potential could consist of identifying laboratories with relatively low diagnostic performance by a penetrator. Moreover, the activation and collaboration procedures inside the network could be disturbed in case of emergent situations including bioterrorism. Yet, the document does not offer this type of information and the established procedures are robust enough not to be disturbed. Moreover, the document defines clear procedures for laboratory response in cross-border events and indicates possible supportive collaboration between laboratories in different Member States. So, the benefit clearly outweighs a probable misuse.

All in all, the following summary, including recommendations, may be given on the outcomes of the JA:

- The degree of laboratory preparedness for the detection of highly pathogenic infectious agents varies at (national and) international level which indicates the need and possibilities for mutual support.
- All participants underlined the usefulness of the EQAEs and could improve their diagnostic capabilities and/or evaluate their high standard.
- The training courses offered significant benefits to trainees and trainers.
- The initiative has collected experiences on biosafety, biosecurity, and transportation issues throughout Europe. During the exercises, no serious problems occurred in terms of transportation due to an intensive preparation of shipment with a neatly selected shipping agency considering all relevant national and international regulations.
- The questionnaire on Biosafety and Biosecurity is offered to the EU for further development and implementation as recommendations for safe and secure handling and exchange of pathogenic material between European Member States and EFTA as well as other countries.
- International proficiency tests for diagnostics of highly pathogenic bacteria are recommended as a continuous process as most EU Member States do not use this instrument at national level.
- A repository of reference material of highly pathogenic infectious agents has been set up and should be maintained on a long-term basis.
- A stable network of laboratories responsible for the diagnostic of highly pathogenic bacteria and viruses is required as these agents also occur with often unknown and underestimated prevalence and could occasionally be imported to EU Member States.
- Common recommendations for the testing of antimicrobial susceptibility of highly pathogenic bacteria and innovative diagnostic methods should be developed for European countries.

Finally, we came to the conclusion that the benefit for all participating laboratories and therefore for the health protection of the citizens was stronger than the minimized residual risk of our activities, which gave us the opportunity to carry out the project from the perspective of DURC assessment. It were taken all measure to minimize the misuse of exchanged biological material, to make these measures transparent to the legal agencies and authorities and to create a network of trusted and reliable laboratories, which professionally handle biological material and information to prevent any misuse of it.

## Conflict of Interest Statement

This presentation has been produced with the support of the European Commission’s Consumers, Health and Food Executive Agency (CHAFEA). Its content is the sole responsibility of the authors and can in no way be taken to reflect the views of the CHAFEA or any other body of the European Union.

## Supplementary Material

The Supplementary Material for this article can be found online at http://www.frontiersin.org/Journal/10.3389/fpubh.2014.00199/abstract
