# Supplementary material for: Benefits of a European Project on Diagnostics of Highly Pathogenic Agents and Assessment of Potential “Dual Use” Issues
Source: Front Public Health. 2014 Nov 11;2:199. doi: 10.3389/fpubh.2014.00199 (PMC4227464; doi:10.3389/fpubh.2014.00199)
Supplement: Supplementary file 1 [file Presentation1.PDF]

Supplementary Material

1: Information on EQAEs

Both networks in framework of the Joint Action QUANDHIP, technically named NIB (Network on Highly Infectious Bacteria) and NIV (Network on highly Infectious Viruses), have performed three External Quality Assurance Exercises (EQAEs). Together with previous activities both networks have collected experiences from 6 EQAEs each.

Exemplarily, sample design and performance of the EQAE are shown for EQAEs on bacteria. The agents were grown on appropriate nutrient media and collected in appropriate amounts for further processing.

For the preparation of usually 10-15 **inactivated samples**, grown bacteria were inoculated into PBS at high concentrations (10^8-10^{10}/ml). The cell number of each bacterial suspension was determined by analysing colony forming units per ml (cfu/ml). The suspensions were inactivated by heat at 60°C for at least 22 h, except for suspensions containing *B. anthracis* which were treated with 1% peracetic acid (PAA) in 80% ethanol for 30 min with subsequent washing. In some cases, the prepared bacterial suspensions were inactivated by 30 kGy gamma-irradiation according to an established standard procedure (20). All inactivated suspensions (pools) were checked for sterility of one tenth of each volume in suitable solid and fluid enrichment media over at least 14 days depending on the agent. After safe inactivation, cell numbers in each suspension were determined using a counting chamber and/or only by real-time PCR. Cross-contamination with other target bacteria was excluded by by specific real-time PCR assays. To increase the challenge, the inactivated bacteria were partially mixed with typical environmental matrices modulating food, outdoor water, or clinical specimens. An example is given in table 1. Usually, 5 samples were indicated as “time crucial”. One of the tasks to be fulfilled by the participants was to identify correctly the target bacteria; in the case of time crucial samples as fast as possible. To verify the time for the latter the secured tailored online mask recorded the time of provided results. In two exercises pure inactivated bacterial samples were used for quantification purposes with the final aim to develop quantitative standards for these bacteria.

The usually 7-15 **living samples** were prepared as pure or mixed cultures with typical bacterial contaminants occurring in real life scenarios. An example is given in table 1. For quality control, the expected growth of the cultures was checked by the provider shortly before sending, then again 2 days after sending and 14 days after sending when the advised

investigation time was expired. The participants were asked to identify highly pathogenic bacteria with their established procedures and methods. The whole panel of possible bacteria was agreed with the participants and defined as so called target bacteria. On an optional basis, participants were asked to perform an antimicrobial susceptibility testing (AST).

Typically 5 samples were indicated as “time crucial”. The tasks for the participants were to identify the target bacteria, in case of time crucial samples as fast as possible. To check the latter approach the secured online mask was registering the time to provision of the results. Participants were explicitly advised to strictly handle these samples under BSL 3 conditions. The accompanying Material Transfer Agreement, which was generally agreed in the framework of the Consortium Agreement, allowed to save the cultures for internal validation purposes but did not allow to pass the samples on to third parties without agreeing the subsequent conditions with the provider.

The following data is based on exemplarily selected 4 comparable EQAEs organized in the framework of NIB over a 5 year period, 2 in the framework of the previous EQADeBa project (E-1; E-2) and 2 in the framework of the on-going QUANDHIP Joint Action (Q-1; Q-2).

The bacterial EQAEs were focused on *B. anthracis*, *Y. pestis*, *F. tularensis*, *B. pseudomallei*, *B. mallei*, *B. melitensis*-group, and only in Q-2 on *C. burnetii*. 7-15 samples, according to the exercise scenario, containing living bacteria (native samples), partially mixed with typical “contaminating” bacteria and inactivated bacteria in a variety of complex matrices and were provided by the RKI. A typical composition of an EQAE with bacteria is given in table 1. A typical example of inactivated samples provided in EQAE Q-1 is given in table 2. A set of quantitatively characterized reference material is listed in table 3.

Table S1 Native samples provided in Q-2

Code	agent I (target bacteria)	agent II (non-target)	Matrices	culture: pure or mixed /expected growth
1	*Burkholderia pseudomallei*	*Pseudomonas aeruginosa*	HCA-softagar	mixed / growth expected
2	*Brucella melitensis*-group (*B. ovis*)	non	cell culture medium	pure / growth expected
3	*Francisella tularensis* ssp. *holarctica*	*Klebsiella pneumoniae*	HCA-softagar	mixed / growth expected very high level of difficulty

4	*Yersinia pestis*	*Pasteurella aerogenes*	HCA-softagar	mixed / growth expected
5	*Bacillus anthracis*	*Staphylococcus aureus*	HCA-softagar	mixed / growth expected high level of difficulty
6	*Francisella tularensis* ssp. *holarctica*	non	cell culture medium	pure/ growth expected
7	*Brucella melitensis*-group (*B. neotomae*)	non	cell culture medium	pure / growth expected
8	*Brucella melitensis*-group (*B. canis*)	*Staphylococcus aureus*	HCA-softagar	mixed / growth expected
9	*Coxiella burnetii*	non	cell culture medium	pure / no growth expected (inactivated sample)
10	*Francisella tularensis* ssp. *mediasiatica*	non	cell culture medium	pure / growth expected

Table S2 Inactivated samples provided in Q-1

**Code**	**Modulated sample**	**Inactivated bacteria / Matrices**	**Number of spiked target-bacteria / ml**
1	Food	*Brucella suis* in milk	5x 10^6
2	Food	*Escherichia coli* in milk	2x 10^7
3	Food	*Bacillus anthracis* in milk	4x 10^5
4	Food	Negative Control matrix milk	0
5	Clinical	*Yersinia pestis* in fetal calf serum	2x 10^7

6	Clinical	*Burkholderia mallei*<br>in fetal calf serum	7x 10^7
7	Clinical	*Francisella tularensis holarctica*<br>in fetal calf serum	1x 10^5
8	Clinical	*Francisella philomiragia*<br>in fetal calf serum	3x 10^7
9	Clinical	*Yersinia enterocolitica*<br>in fetal calf serum	9x 10^7
10	Clinical	Negative Control<br>matrix fetal calf serum	0

Table S3 Inactivated reference material Q-2

Code	Inactivated bacteria in PBS	Spiked target-bacteria in GE/ml (mean value from TaqMan results)
1	*Francisella tularensis* ssp. *tularensis*	4,5 x 10^9
2	*Yersinia pestis*	1,5 x 10^8
3	*Bacillus anthracis* (approx. 80% spores)	2,8 x 10^5
4	*Francisella tularensis* ssp. *holarctica*	4,7 x 10^9
5	*Burkholderia mallei*	5,9 x 10^8
6	*Burkholderia pseudomallei*	6,7 x 10^8
7	*Brucella melitensis*- group (*B. melitensis*)	1,2 x 10^9

Shipment

A market analysis revealed a provider who could be identified as a reliable shipper in terms of shipment quality, including management and Biosafety and Biosecurity.

Table 4 shows the mean transportation time of samples for 4 exercises (E-1 is excluded because only inactivated samples were shipped).

Table S4 Transportation time for samples of 4 EQAEs

EQAE	N participants	Mean transport time (h)	Mean deviation of transport time (h)
E-2	21	29.2	18.1
E-3	21	29.6	16.8
Q-1	28	29.8	14.6
Q-2	28	25.7	8.0

It can be concluded that the time required for delivery of samples was quite stable over the exercises with a tendency for reduction in Q-2. There is also a tendency for reduction of the time deviation which means that the participants received their samples in a closer time slot. All in all, the transportation time was relatively short and border issues were an exception and could be solved without serious problems.

Response time of laboratories - Submission of time critical results

During each of the exercises mentioned 5 living/native samples were "time critical" which means that the participants should provide their results as soon as possible (table 5).

Table S5: First response time of the participants to time critical samples

**EQAE**	**N participants**	**Mean response time (h)**	**Mean deviation of response time (h)**
E-2	20	15.3 (29.3)	15.4 (24.7)
E-3	20	11.5 (20.6)	11.8 (29.3)
Q-1	28	8.4 (12.3)	8.8 (12.8)
Q-2	28	5.7 (9.1)	3.8 (7.4)

In brackets: Second response time including the differentiation of subspecies (*F.tularensis*) and species (*Brucella*)

Usually, the analyses were done in two steps: first, a screening step for identification at genera and species level, and second, a further identification at species (e.g. *Brucella*) and subspecies (e.g. *Francisella tularensis*) level, where indicated (table 5).

It becomes very obvious that the involved laboratories substantially improved their response time by about 70 %. This important improvement is due to a better preparedness of the laboratories through training effects during the exercises and an improvement of diagnostic algorithms and methods.

Submission of correct qualitative results

The submission of correct results and the assessment of good performance of the laboratories is a major element of EQAEs. The following tables 6 and 7 will give an overview on the correct analyses of living and inactivated samples.

Table S6 Diagnostic of Native Samples

		Correct Results on Native Pathogens in %						
**Joint Action**	**EQAE** (n participants)	**B.a.** Corr pos	**Y.p.** Corr pos	**F.t.ssp**[1] Corr pos (Species level)	**Bruc.sp**[3] Corr pos (Genus level)	**B.m.** Corr pos	**B.psm** Corr pos	**Negative control** Corr neg
**EQADeBa**	E2 (n=21)	90	86	73 (88)	86 (95)	86	90	91
	E3 (n=22)	100	96	86 (100)	89 (96)	96	96	98
**QUANDHIP**	Q1 (n=28)	96	--	--	89 (98)	--	96	84[5]
	Q2 (n=27)	93	93	72[2] (92)	58[4] (94)	--	96	--
**mean**		**95**	**92**	**77 (93)**	**81 (96)**	**91**	**95**	**91**

[1] *F.t.* only correct subspecies was rated; in brackets correct at species level rated
[2] *F.t.* ssp. *mediasiatica* difficult to identify
[3] *Brucella* only correct species was rated; in brackets correct as *B. melitensis* group rated
[4] Three *Brucella* species had to be differentiated
[5] *F.t.* ssp. *novicida* was quite frequently detected as target bacteria
Abbreviations: B.a.- *Bacillus anthracis*; Y.p.- *Yersinia pestis*; F.t. - *Francisella tularensis*; Bruc.- *Brucella*; B.m. – *Burkholderia mallei*; B.psm. – *Burkholderia pseudomallei*; Corr pos – Correct positive; Corr neg – Correct negative

Table S7 Diagnostic of Inactivated Samples

		Correct Results on Inactivated Pathogens in %						
	EQAE (n participants)	B.a. Corr pos	Y.p. Corr pos	F.t. ssp[1] Corr pos (Species level)	Bruc. sp[2] Corr pos (Genus level)	B.m. Corr pos	B.psm. Corr pos	Negative Corr neg
**EQADeBa**	E1 (n=21)	94	84	33 (97)	--	--	--	84
	E2 (n=24)	96	92	67 (92)	73 (92)	88	88	93
	E3 (n=23)	91	94	87 (100)	83 (87)	94	89	97
**QUANDHIP**	Q1 (n=29)	90	83	76 (97)	55[3] (97)	90	--	95
	Q2 (n=29)	90	86	83	62 (83)	93	76	--
**mean**		**92**	**88**	**69 (94)**	**68 (90)**	**91**	**84**	**92**

[1] *F.t.* only correct subspecies was rated; in brackets correct on species level rated
[2] *Brucella* only correct species was rated; in brackets correct as *B. melitensis* -group rated
[3] Identification of *Brucella suis* failed
Abbreviations: B.a.- *Bacillus anthracis*; Y.p.- *Yersinia pestis*; F.t. - *Francisella tularensis*; Bruc.- *Brucella*; B.m. – *Burkholderia mallei*; B.psm. – *Burkholderia pseudomallei*; Corr pos – Correct positive; Corr neg – Correct negative

As mentioned above, an additional challenge consisted in the fact that targets were partially mixed with complex matrices.

In addition Q-2 (n=26) also included *C. burnetii*; laboratories provided 96% correct identification.

In addition, even more profound analyses have been performed to identify best practices for correct and to reveal reasons for incorrect results (data not shown), which were discussed with the participants. This included applied algorithms and methods. Important conclusions could be drawn and related recommendations for improvement could be given, where appropriate.

As a conclusion, PCR or immunological approaches were identified as best practices for sample analyses as a first step for preliminary identification of target bacteria which should be followed by a confirmation by cultivation/isolation of bacteria and subsequent identification of growing germs by PCR or other applicable methods like MALDI-TOF.

Submission of correct quantitative results

To our knowledge, there are no quantitative standards available for highly pathogenic bacteria. Therefore, we aimed to produce relevant reference material of all target bacteria in a standardized procedure. The material has been produced in sufficient amounts and is properly stored so that it can be provided as quantitative standards for quite a long time. Beyond the assessment of quantification of samples by different partners, the EQAEs were also used to generate a consensus quantitative mean for these samples. Table 8 exemplarily shows results on quantification obtained in Q-2.

Table S8 Quantitative Analyses of Inactivated Reference Samples in Q-2

**log10 GE/ml**	**Pathogen**							
	B.a.	Y.p.	F.t.h.	F.t.t.	B.melit.	B.m.	B.psm.	C.burn.
**Mean**	5.7	4.0	6.2	6.2	6.2	5.6	5.3	7.1
**Reference**	5.9	6.3	7.1	7.0	6.1	5.7	5.3	6.8
**Range**	3.5	3.9	3.8	3.7	4.4	3.1	3.9	5.0

10-16 participants, depending on the pathogen, provided results on quantification.
“Reference” means data obtained by the provider (RKI).
Abbreviations: B.a.- *Bacillus anthracis*; Y.p.- *Yersinia pestis*; F.t.h - *Francisella tularensis* ssp. *holarctica*; F.t.t - *Francisella tularensis* ssp. *tularensis*; B.melit.- *Brucella melitensis*; B.m. – *Burkholderia mallei*; B.psm. – *Burkholderia pseudomallei*; C.burn – *Coxiella burnetii*; Corr pos – Correct positive; Corr neg – Correct negative

From this study it can be concluded that the range of results on the quantitative reference samples is by far too high. This could have several reasons including different methods, sensitivity of applied methods, different standards, different calculations etc. Thus, together with appropriate quantitative standards, standard operational protocols are required to obtain comparable results.

All in all, it can be concluded that the laboratories performed on a high level of diagnostic quality. However, if the sample composition varied and got more complex and challenging, the overall and individual results became worse, as seen during Q-2 and further extended during Q-3 (data not shown). Problems occurred with the correct identification of *Brucella* species and *Francisella tularensis* subspecies. Mixed samples were a challenge for several laboratories and a further optimization is required when applying selective media to isolated target bacteria for the confirmation of the diagnosis. In some cases sensitivity and specificity of the applied PCR are not sufficient and need further optimization. Quantification of target bacteria is a real issue which has to be improved by future activities. The antimicrobial susceptibility testing should be further improved and new methods for diagnostics of highly pathogenic bacteria, such as MALDI-TOF and hand-held test kits, should be further validated (data not shown). Topical working groups were set up in the framework of NIB profoundly tackling AST, MALDI-TOF and the development of quantitative reference materials. Regarding AST, the European Committee on Antimicrobial Susceptibility Testing (EUCAST) has been interested in our activities and a close contact was established with the aim to approve our results and take those into consideration for the development of appropriate European standards.

2: Material Transfer Agreement template used in QUANDHIP as part of the Consortium Agreement

The RKI acknowledges that the Materials are or may be the subject of a patent application. No proprietary rights or licenses or other rights are granted by this agreement.

The RKI acknowledges that the Materials will be transferred, distributed to third parties only for the restricted purpose mentioned above.

The Materials provided are experimental in nature. They are provided without warranty of merchantability or fitness for a particular purpose. The Provider makes no representation that the use of the Materials will not infringe any patent, copyright, trademark or other proprietary right.

The Provider affirms that he is authorised for providing the Materials to the RKI for the purpose mentioned above.

Upon the request the RKI shall promptly return to the Provider the Materials furnished to RKI under this agreement.

Except to the extent prohibited by law, RKI assumes all liability for damages or injury resulting from the use, storage or disposal of the Materials. RKI agrees hereby to hold harmless the Provider, its staff members, employees and agents from any loss, claim, damage, expense or liability, of whatsoever kind of nature (including attorney's fees), which may arise from or in connection with this Agreement or the use, handling or storage of the Materials.

If any provision of this agreement shall be found by a court of competent jurisdiction to be void, invalid or unenforceable, the same shall either be reformed to comply with applicable law or stricken if not so conformable, so as not to affect the validity or enforceability of this agreement.

Amendments and modifications to the text of this Material Transfer Agreement (MTA) require a separate written agreement between the RKI and the Provider. This MTA shall be construed in accordance with and governed by the laws of Luxembourg. Place of jurisdiction shall be Luxembourg.

The costs for the transport of the Material will be paid by the Provider (Partner).

Signed by duly authorized representatives of RKI and Recipient and acknowledged by responsible Scientists

PROVIDER

Signature: ....

Name (block letters): ....

Position:...

Date: ....

ROBERT KOCH-INSTITUT

Signature:...

Name (block letters):......................................

Position: ....

Date:..............................

Scientist in charge of the Project:

Name: ....

Signature: ....

Date: ....

Scientist in charge of the Project:

Dr. Roland Grunow

Signature: ....

Date: ....

The RKI acknowledges that the Materials are or may be the subject of a patent application. No proprietary rights or licenses or other rights are granted by this agreement.

The RKI acknowledges that the Materials will be transferred, distributed to third parties only for the restricted purpose mentioned above.

The Materials provided are experimental in nature. They are provided without warranty of merchantability or fitness for a particular purpose. The Provider makes no representation that the use of the Materials will not infringe any patent, copyright, trademark or other proprietary right.

The Provider affirms that he is authorised for providing the Materials to the RKI for the purpose mentioned above.

Upon the request the RKI shall promptly return to the Provider the Materials furnished to RKI under this agreement.

Except to the extent prohibited by law, RKI assumes all liability for damages or injury resulting from the use, storage or disposal of the Materials. RKI agrees hereby to hold harmless the Provider, its staff members, employees and agents from any loss, claim, damage, expense or liability, of whatsoever kind of nature (including attorney's fees), which may arise from or in connection with this Agreement or the use, handling or storage of the Materials.

If any provision of this agreement shall be found by a court of competent jurisdiction to be void, invalid or unenforceable, the same shall either be reformed to comply with applicable law or stricken if not so conformable, so as not to affect the validity or enforceability of this agreement.

Amendments and modifications to the text of this Material Transfer Agreement (MTA) require a separate written agreement between the RKI and the Provider. This MTA shall be construed in accordance with and governed by the laws of Luxembourg. Place of jurisdiction shall be Luxembourg.

The costs for the transport of the Material will be paid by the Provider (Partner).

Signed by duly authorized representatives of RKI and Recipient and acknowledged by responsible Scientists

**PROVIDER**	**ROBERT KOCH-INSTITUT**
Signature:	Signature:..
Name (block letters):	Name (block letters):....................................
Position:..	Position: ....
Date:	Date:...........................
Scientist in charge of the Project:	Scientist in charge of the Project:
Name:	Dr. Roland Grunow
Signature:	Signature: ....
Date:	Date: ....
